# The Impact of Sex and Age on Antipsychotic Serum Concentrations

**DOI:** 10.1093/schbul/sbaf217

**Published:** 2026-03-21

**Authors:** Franciska de Beer, Bodyl A Brand, Ben Wijnen, Shiral S Gangadin, Georgios Schoretsanitis, Daan Touw, Iris E C Sommer

**Affiliations:** Center for Clinical Neuroscience and Cognition, University Medical Center Groningen, University of Groningen, Groningen, 9713 AP, The Netherlands; Department of Psychiatry, University of Oxford, Oxford, OX3 7JX, United Kingdom; Centre of Economic Evaluations & Machine Learning, Trimbos Institute, Netherlands Institute of Mental Health and Addiction, Utrecht, 3521 VS, The Netherlands; Center for Clinical Neuroscience and Cognition, University Medical Center Groningen, University of Groningen, Groningen, 9713 AP, The Netherlands; Rob Giel Research Center, University Center Psychiatry, University Medical Center Groningen, University of Groningen, Groningen, 9713 GZ, The Netherlands; Department of Psychiatry, Psychotherapy and Psychosomatics, Hospital of Psychiatry, University of Zurich, Zurich, 8008, Switzerland; Department of Psychiatry, Northwell Health, The Zucker Hillside Hospital, Glen Oaks, NY, 11004, United States; Department of Clinical Pharmacy and Pharmacology, University Medical Center Groningen, University of Groningen, Groningen, 9713 GZ, The Netherlands; Department of Pharmaceutical Analysis, Groningen Research Institute of Pharmacy, University of Groningen, Groningen, 9713 AV, The Netherlands; Center for Clinical Neuroscience and Cognition, University Medical Center Groningen, University of Groningen, Groningen, 9713 AP, The Netherlands

**Keywords:** antipsychotic medication, sex, menopause, therapeutic drug monitoring, psychosis

## Abstract

**Background and Hypothesis:**

Estrogen affects drug metabolism for specific antipsychotics, which may produce sex differences in serum levels, especially over the menopausal transition. Here, we explore sex differences in concentrations of four commonly used antipsychotics: clozapine, olanzapine, aripiprazole, and quetiapine and examined how these vary across age groups, including postmenopause (>55).

**Study Design:**

Antipsychotic concentrations of 44 378 serum samples drawn between January 2016 and October 2024 were analyzed. Samples were provided by 6147 unique adult men and women for clozapine (*n* = 34 761 samples, 26% female), olanzapine (*n* = 5114, 27% female), aripiprazole (*n* = 3171, 37% female), and quetiapine (*n* = 1332, 50% female). To investigate the effect of sex and age on antipsychotic concentrations, we employed linear mixed-effects models and pairwise contrasts by dividing the samples in 6 groups according to sex and age (<45, 45-55, and >55 years).

**Study Results:**

We found higher concentrations in women compared to men for clozapine (*P* < .001), and olanzapine (*P* < .001), but not for quetiapine or aripiprazole. Young women (<45) showed higher levels of clozapine (*P* = .004) and olanzapine (*P* < .001) than men, but older women did not. Clozapine levels were higher in women <45 and 45-55 compared to women >55 years (all *P* < .05). For olanzapine, aripiprazole, and quetiapine concentrations differences between female age groups were absent.

**Conclusions:**

We found sex differences in olanzapine and clozapine concentrations, which were most pronounced until 45 years of age. Our findings suggest that monitoring antipsychotic levels may add clinical value and underscore the need for sex-specific prescription guidelines for olanzapine and clozapine.

## Introduction

Antipsychotic prescriptions and titrations seldom consider sex,^1-3^ although some guidelines do recommend lower clozapine dosing for non-smoking women.^4^ Yet, robust sex-related differences are found in antipsychotic efficacy and tolerability.^5^^,^^6^ Although their primary indication remains schizophrenia and schizophrenia-related disorders, most antipsychotic drugs are also used to treat a broad range of symptoms and disorders, including bipolar mania and depression, unipolar depression that is unresponsive to standard antidepressant treatment, Tourette’s disorder, and irritability associated with autism spectrum disorder.^7^ In terms of efficacy, young women tend to respond better to antipsychotic treatment, but appear to lose this advantage around the age of 45,^8-10^ as a recent study showed that the effectiveness of antipsychotics in preventing relapse decreases around that age in women, but not in men.^11^ In terms of tolerability, women are more susceptible to experience adverse reactions,^12-16^ with female sex being the second most important risk factor for severe side-effects of antipsychotics, after polytherapy.^17^ Being female (ie, sex assigned at birth) has repeatedly been shown to correlate with more severe metabolic,^18-22^ extrapyramidal,^23^ and endocrine side effects, including female-specific health problems such as menstrual dysfunction.^24-26^ The risk for side effects becomes particularly relevant in women around the time of menopause, as menopause itself brings health risks that strongly overlap with the side effects of long-term antipsychotic use, including obesity and diabetes, cardiovascular disease, and osteoporosis.^27^

The increased efficacy of antipsychotics in young women, together with their higher susceptibility to side effects, may be related to pharmacokinetic differences. With equal dosing, women may show higher serum concentrations than men due to slower absorption, metabolism, and excretion. Women secrete less gastric acid, have slower gastric emptying and prolonged colonic transit, smaller kidneys and liver, and differences in protein binding, all affecting drug disposition.^12^

Many of these pharmacokinetic mechanisms are influenced by the ovarian hormones estrogen and progesterone.^12^^,^^15^ Both estrogens and progesterone influence gastric emptying and act on p-glycoprotein, an efflux transporter expressed in the gut, liver, kidneys, and blood brain barrier, affecting drug absorption, transport into the brain, and elimination.^28^^,^^29^ Progesterone prolongs colonic transit time, which increases the absorption rate in women,^30^ whereas estrogens impact absorption by reducing gastric acid production.^29^^,^^31^ In the liver, estrogens influence the elimination of specific antipsychotics by acting on several main enzymes responsible for first-pass phase I metabolism of antipsychotics.^12^^,^^32^^,^^33^ On the one hand, estrogens *inhibit* cytochrome P450 (CYP) enzymes 1A2 and 2C19, while on the other hand, estrogens *induce* the activity of CYP3A4, and to a lesser extent of CYP2D6.^12^^,^^32^^,^^34^ Every antipsychotic is metabolized by a different enzyme profile,^32^ hence sex differences may be antipsychotic-specific. For example, olanzapine is metabolized primarily by CYP1A2 and UGT1A4, clozapine by CYP1A2 and CYP2C19, whereas quetiapine is predominantly metabolized by CYP3A4 and aripiprazole by CYP3A4 and CYP2D6.^32^

Given pivotal role of estrogen on drug pharmacokinetics, periods of hormonal fluctuations such as perimenopause, the transition to menopause, may impact antipsychotic serum concentrations and thus the tolerability and efficacy of antipsychotics. Indeed, two therapeutic drug monitoring (TDM) studies have shown higher antipsychotic serum concentrations of most antipsychotics in women as compared to men.^35^^,^^36^ One TDM study indicated that higher dose-corrected plasma concentrations of quetiapine become particularly evident above the age of 40,^36^ and another study showed that sex differences in dose-adjusted concentrations of paliperidone proportionally doubled after the age of 50, again being the highest in women after the age of 50.^37^

Although sex and hormonal life stages may influence antipsychotic concentrations, research on these effects remains limited. This study examines how serum concentrations of clozapine, olanzapine, aripiprazole, and quetiapine differ between sexes and evolve over different age groups related to pre-, peri-, and postmenopausal stages.

## Methods

### Patients and Samples

**Figure 1:**
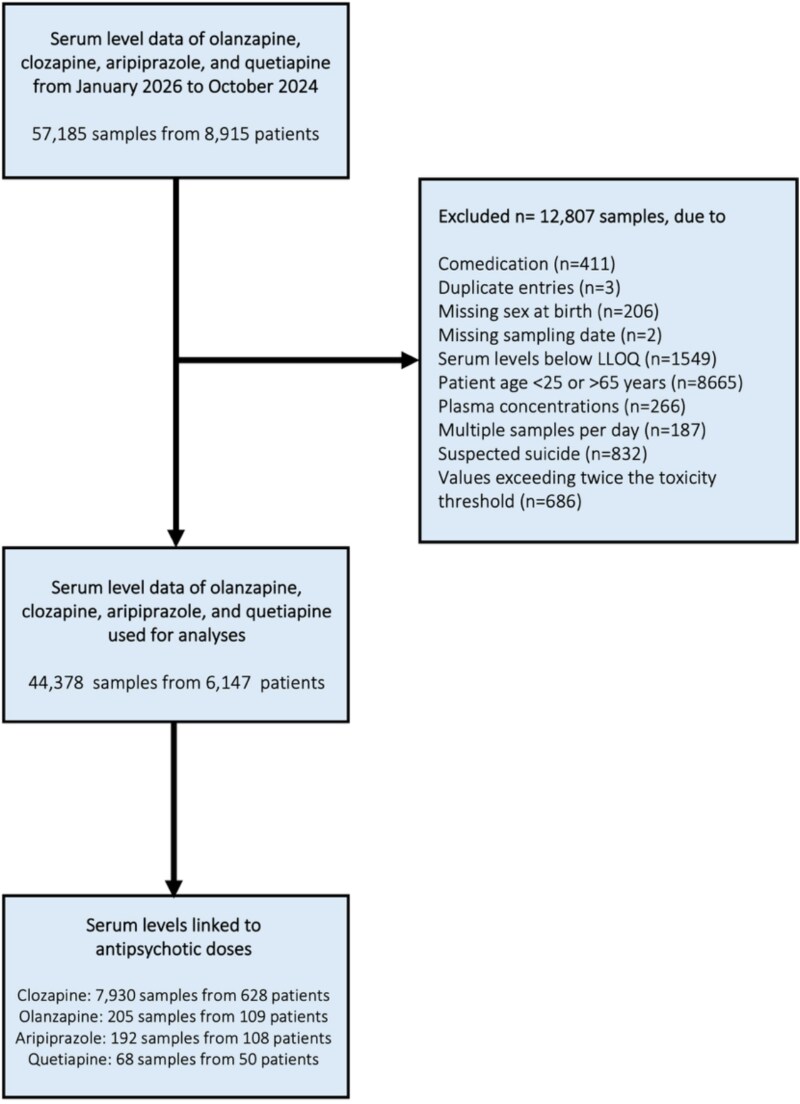
Flowchart of Patient Inclusion and Exclusion.

Antipsychotic serum concentrations were collected from the laboratory information system (GLIMS) database from the department of Clinical Pharmacy and Pharmacology of the University Medical Center Groningen (UMCG), the Netherlands. From this database, serum level data was extracted for clozapine, olanzapine, aripiprazole, and quetiapine. Analyses of additional antipsychotics were not feasible, due to limited sample sizes per sex. Data were collected between January 2016 and October 2024 and included serum concentrations, anonymized patient identifier, date of birth, sex, and the date and time of measurement. Serum levels were predominantly measured in the morning, with comparable average sampling times between male and female patients across all antipsychotics. Data were excluded from the analyses if the patient was younger than 25 or older than 65 years at the moment of measurement; if suicide by overdose was suspected, identified by multiple serum measurements within 7 days, with at least one exceeding laboratory alert levels; if serum levels exceeded twice the toxicity threshold.^32^ if serum levels were below the lower limit of quantification (LLOQ); if the patient also had a measurement of duloxetine, paroxetine, fluoxetine, fluvoxamine, or venlafaxine within the same month, as these drugs are known to affect antipsychotic serum levels.^38^ A flowchart illustrating the inclusion and exclusion of patients and samples is provided in Figure 1.

### Serum Analyses

For the antipsychotic drugs, the serum levels were determined in the laboratory of the department of Clinical Pharmacy and Pharmacology in the UMCG (ISO15189 accredited) via routine analytical methods. Plasma samples were analyzed with a UPLC-MSMS technique (Thermo Vanquish® and Quantiva® UPLC-MSMS system, equipped with a Thermo Accucore® C18 column, all from Thermo, USA). Samples were prepared by making a mixture of 100 μL plasma and 500 μL of an internal standard solution containing a stable isotope of the drug and 0.04 mg/L cyanoimipramine in methanol, and centrifuging this at 10 000 rpm for 5 min. 0.5 μL of the supernatant was then injected into the UPLC-MSMS system. A gradient elution method was employed at a flow rate of 1.0 mL/min with an initial mobile phase composition of 0.02 mol/L ammoniumformate buffer (pH 3.5) in Millipore water/methanol (65/35). This ratio transitioned to 5/95 by 1.15 min, followed by re-equilibration to the initial conditions to clean the column for subsequent analyses. The method demonstrated linearity for analyte concentrations ranging from 5 to 500 μg/L for all drugs, covering the expected serum concentration range (50-450 μg/L). The LLOQ was 5 μg/L for all drugs, with intra-day and inter-day precision being lower than 15% for low, medium, and high concentrations. All reagents used were of analytical grade. Reference compounds were purchased from Sigma (the Netherlands), and stable isotopes were purchased from Alsachim (France).

### Antipsychotic Doses

Antipsychotic doses were available for a sub-group of the cohort sample. These were individuals who also participated in the Pharmacotherapy Monitoring and Outcome Survey (PHAMOUS).^39^ In order to compute dose-corrected serum concentrations, we linked blood samples from the Clinical Pharmacy and Pharmacology Department in the UMCG to PHAMOUS Routine Outcome Monitoring data. PHAMOUS, a subcohort of MindLines,^40^ is an ongoing naturalistic dynamic cohort of individuals with psychotic disorders and/or current antipsychotic use who receive care in one of four participating mental healthcare facilities in the Northern Netherlands: GGZ Drenthe, GGZ Friesland, Lentis Psychitric Institute, and University Center Psychiatry (UCP) Groningen. In accordance with the declaration of Helsinki, these service users are informed that their pseudonymized data could be used for scientific research, and they have the option to opt out of data use. Research on this dataset has been deemed exempt from the Medical Research Involving Human Subjects Act (WMO) by the Medical Ethical Committee of the UMCG (METc 2025/298. research register number: 21394). This exemption was granted because no additional burden is placed on service users, the research involves pre-existing routine healthcare data, and research on the data serves a broad public interest.

As part of the annual monitoring survey, current medication use is recorded. For this study, we included observations collected between 2016 and 2022. Dose data were linked to serum concentration data if the blood draw occurred within 1 year after the monitoring survey. Linked dose-concentration data was available for clozapine (*n* = 651 unique patients), olanzapine (*n* = 118), aripiprazole (*n* = 115), and quetiapine (*n* = 52) (Appendix B). Concentration-to-dose (C/D) ratios are presented descriptively (Appendix B) to aid in the interpretation of the LMEM results and to evaluate whether observed sex differences in serum concentrations were attributable to differences in prescribed doses or to underlying pharmacokinetic variation.

### Statistical Analysis

Samples were grouped by age into the following categories: <45 years (premenopausal), 45-55 years (perimenopausal), and >55 years (postmenopausal). To test whether antipsychotic serum levels differ between men and women for each age group, linear mixed effects models (LMEM) were used with antipsychotic serum concentration as dependent variable, random intercept for participants, and fixed effects for sex, age in weeks, menopausal age group, and the interaction term between sex and menopausal age group. LMEM allow for repeated observations and assume each patient had a different starting point (random intercept) and that patients can be affected by the fixed factors of age, sex, and age group. Next, using estimated marginal means, 6 a priori defined contrasts were tested: 3 comparing men and women within each age group (<45, 45-55, and > 55 years), and 3 comparing women across age groups (<45 vs 45-55 years, 45-55 vs >55 years, and <45 vs >55 years). Statistical analyses were performed in R via Rstudio (version 4.3.2).^41^

## Results

Analyses included a total of 44 378 samples from 6147 patients of which 35% (*n* = 2147) were female with a mean age of 44 (SD 11.49) years. Key demographic data for the patients including age and mean serum concentrations can be found in Table 1.

**Table 1:** Patients and Samples Characteristics

				**Male**			**Female**		
	**Overall**	**Male**	**Female**	**25-45 years**	**45-55 years**	**55-65 years**	**25-45 years**	**45-55 years**	**55-65 years**
**Overall**									
Number of patients (%)	6147	4000 (65.1%)	2147 (34.9%)	1793 (29.2%)	1596 (26%)	653 (10.6%)	649 (10.6%)	892 (14.5%)	623 (10.1%)
Number of samples (%)	44 378	34 042 (76.7%)	10 336 (23.3%)	15 052 (33.9%)	13 938 (31.4%)	5052 (11.4%)	3085 (7%)	4526 (10.2%)	2725 (6.1%)
Age, mean (SD)	43.8 (11.5)	42.2 (11.1)	46.7 (11.6)	31.9 (4.3)	47.0 (4.6)	60.0 (2.8)	31.9 (4.3)	48.0 (4.4)	60.3 (2.9)
Number of measurements per person, median (SD)	17.7 (32.3)	20.5 (36.1)	12.3 (22.4)	22.9 (39.3)	19.6 (34.7)	16 (28.7)	13.8 (27.4)	12.4 (21.3)	10.6 (17.8)
**Clozapine**									
Number of patients (%)	2548	1880 (73.8%)	668 (26.2%)	829 (32.5%)	775 (30.4%)	276 (10.8%)	209 (8.2%)	293 (11.5%)	166 (6.5%)
Number of samples (%)	34 761	27 622 (79.5%)	7139 (20.5%)	12 105 (34.8%)	11 464 (33%)	4053 (11.7%)	2246 (6.5%)	3163 (9.1%)	1730 (5.0%)
Age, mean (SD)	43.1 (11.4)	42.2 (11.1)	45.7 (11.6)	31.8 (4.5)	46.9 (4.6)	60.3 (2.9)	31.4 (4.3)	47.8 (4.4)	60.2 (3.0)
Concentration (μg/L) of all measurements, mean (SD)	400.2 (209.8)	398.1 (209.7)	408.1 (209.9)	408.0 (219.0)	387.7 (200.8)	398.3 (204.4)	414.9 (219.1)	406.6 (203.3)	402.0 (209.6)
Concentration (μg/L) of first measurement, mean (SD)	321.9 (229.3)	314.1 (224.9)	343.9 (239.9)	301.1 (226.6)	328.6 (225.3)	312.5 (217.1)	335.8 (246.4)	347.7 (240.5)	347.3 (231.4)
Number of measurements per person, median (SD)	32.1 (39.8)	34.3 (42.6)	25.9 (29.7)	39.0 (47.3)	31.1 (38.6)	29.0 (36.6)	29.5 (35.6)	24.4 (26.6)	24.1 (26.4)
**Olanzapine**									
Number of patients (%)	2241	1490 (66.5%)	751 (33.5%)	698 (31.1%)	536 (23.9%)	256 (11.4%)	200 (8.9%)	292 (13%)	259 (11.6%)
Number of samples (%)	5114	3740 (73.1%)	1374 (26.9%)	1769 (34.6%)	1360 (26.6%)	611 (11.9%)	368 (7.2%)	515 (10.1%)	491 (9.6%)
Age, mean (SD)	44.1 (11.9)	42.0 (11.3)	48.2 (11.7)	31.7 (4.1)	47.1 (4.5)	59.6 (2.7)	32.1 (4.3)	48.3 (4.4)	60.6 (2.8)
Concentration (μg/L) of all measurements, mean (SD)	36.1 (25.1)	34.9 (23.6)	39.3 (28.4)	34.6 (23.1)	36 (23.9)	33.6 (24.3)	40.6 (28.8)	39.0 (26.8)	38.6 (29.7)
Concentration (μg/L) of first measurement, mean (SD)	33.8 (25.3)	31.6 (23.1)	38.3 (28.6)	30.5 (21.7)	32.7 (24.3)	32.2 (24.4)	39.3 (31)	37.7 (27.1)	38.1 (28.3)
Number of measurements per person, median (SD)	8.5 (23.0)	9.6 (25.9)	6.3 (15.6)	10.9 (27.9)	8.9 (26.2)	7.3 (18.5)	6.9 (21.1)	6.6 (15.9)	5.4 (9.1)
**Aripiprazole**									
Number of patients (%)	1369	806 (58.9%)	563 (41.1%)	406 (29.7%)	315 (23%)	85 (6.2%)	185 (13.5%)	246 (18%)	132 (9.6%)
Number of samples (%)	3171	2013 (63.5%)	1158 (36.5%)	1004 (31.7%)	780 (24.6%)	229 (7.2%)	319 (10.1%)	562 (17.7%)	277 (8.7%)
Age, mean (SD)	42.7 (10.9)	40.8 (10.4)	45.3 (11.0)	32.3 (4.3)	46.7 (4.7)	59.9 (2.8)	32.2 (4.3)	47.4 (4.3)	59.55 (2.7)
Concentration (μg/L) of all measurements, mean (SD)	192.2 (136.6)	182.4 (132.6)	209.3 (141.8)	165.0 (117.5)	200.3 (143)	197.3 (148.4)	198.4 (137.5)	216.2 (141.1)	207.86 (147.6)
Concentration (μg/L) of first measurement, mean (SD)	189.9 (143.5)	182.4 (135.3)	200.8 (154)	175.5 (127.7)	189.2 (139.1)	189.4 (154.9)	183.9 (140.1)	212.3 (156.9)	202.86 (165.7)
Number of measurements per person, median (SD)	11.1 (24.5)	13.1 (27.9)	8.1 (18.3)	14.7 (30)	12.0 (27)	9.6 (19.8)	8.4 (22.9)	8.7 (17.6)	6.54 (10.6)
**Quetiapine**									
Number of patients (%)	731	335 (45.8%)	396 (54.2%)	112 (15.3%)	140 (19.2%)	83 (11.4%)	110 (15%)	165 (22.6%)	121 (16.6%)
Number of samples (%)	1332	667 (50.1%)	665 (49.9%)	174 (13.1%)	334 (25.1%)	159 (11.9%)	152 (11.4%)	286 (21.5%)	227 (17%)
Age, mean (SD)	46.9 (11.3)	46 (11)	47.7 (11.6)	33.3 (4.2)	47.9 (4.5)	60 (2.8)	32.2 (4.4)	48.6 (4.4)	60.7 (2.9)
Concentration (μg/L) of all measurements, mean (SD)	205.1 (253.5)	231.5 (275.4)	178.6 (226.6)	174.4 (206.8)	260.8 (280.3)	232.5 (319.3)	171.6 (234.7)	160.8 (217.6)	205.7 (230.6)
Concentration (μg/L) of first measurement, mean (SD)	195.1 (290.6)	213.9 (331.2)	179.1 (250.5)	140.7 (188.7)	245.3 (368.8)	259.7 (396.0)	184.9 (262.2)	160.4 (256.2)	199.4 (231.2)
Number of measurements per person, median (SD)	8.2 (20.7)	9.8 (27.1)	6.7 (12.9)	8 (17.1)	13.5 (37.7)	6.2 (11.8)	5.4 (8.1)	6.8 (14.5)	7.83 (14.0)

Age is reported of the first measurement per patient. Therapeutic reference ranges per antipsychotic are: aripiprazole 100-350 ng/mL; clozapine 350-600 ng/mL; olanzapine 20-80 ng/mL; quetiapine 100-500 ng/mL.^32^

### Clozapine

**Figure 2:**
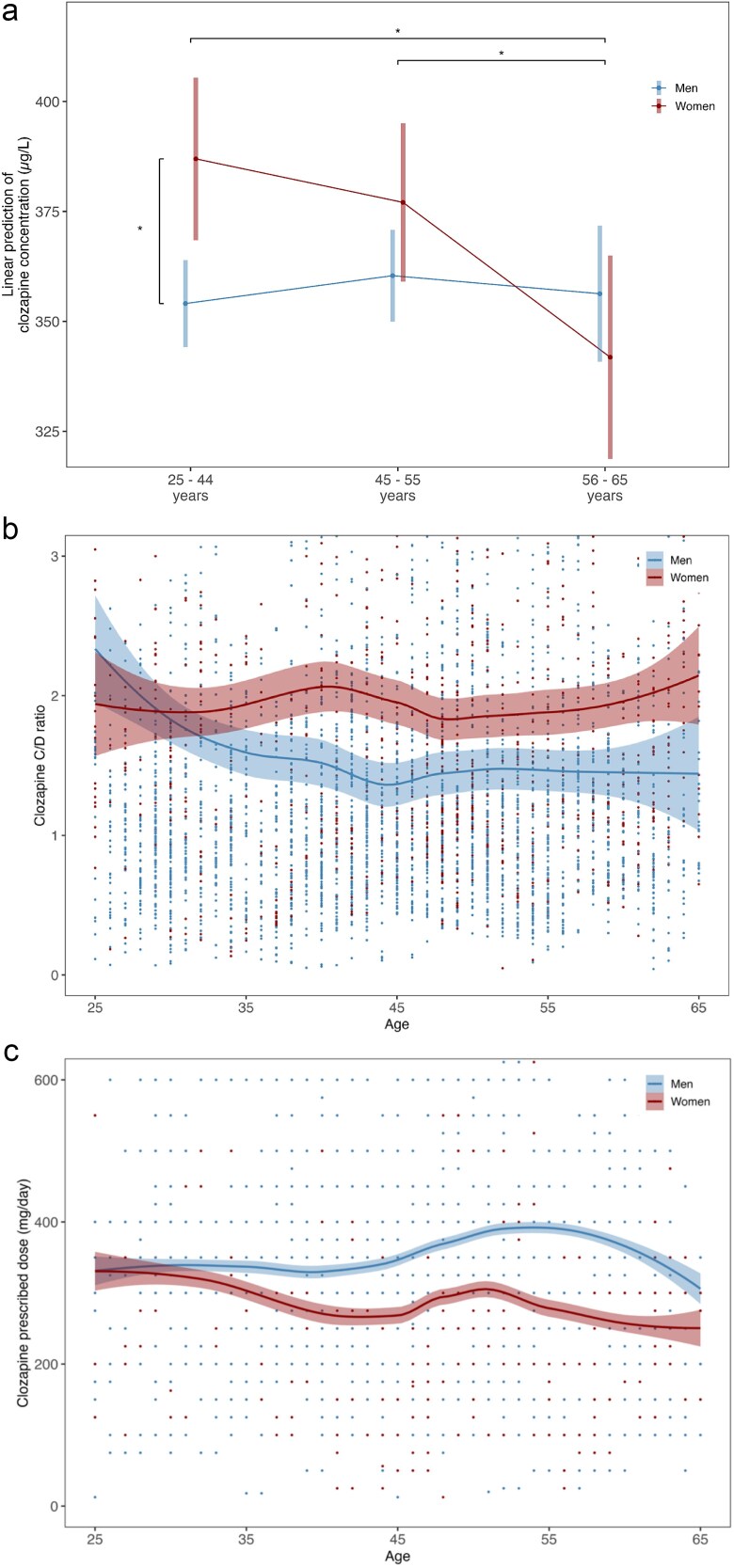
(A) Estimated Marginalized Means (SE) of Clozapine Serum Concentrations (μg/L), Corrected Repeated Measurements and the Overall Age-Related Increase in Concentrations, for Men (27 622 samples of 1880 men) and Women (7139 samples of 668 women) in the Age Groups of <45, 45-55, and >55 years. (B) Clozapine concentration-to-dose (C/D) ratios for men (5906 samples of 457 men) and women (2024 samples of 171 women) across ages, uncorrected for repeated measurements. (C) Clozapine prescribed dose (mg/day) for men and women across ages.

A total of 34 761 samples were analyzed from 2548 patients, of whom 26% (*n* = 668) were female. Clozapine concentrations increased with age (estimate = 0.03, CI = 0.02 to 0.05, *P* < .001, Figure 2A). Women had significantly higher clozapine concentrations than men (patient-aggregated mean 372.13, SD 189.36 for women; 349.81, SD 183.42 for men; estimate = 32.90, CI = 12.49 to 53.30, *P* = .002), as well as higher dose-corrected concentrations (C/D ratios) across all ages (Figure 2B, Appendix B). In both sexes, prescribed clozapine doses decline after age 55 (Figure 2C, Appendix B).

Compared to men of the same age, young women (<45) had significantly higher clozapine concentrations (estimate = 32.898, CI = 5.432 to 60.364, *P* = .004), while no sex difference was observed at age 45-55 years (estimate = 16.661, CI = −10.748 to 44.069, *P* = .163) and >55 years (estimate = −14.440, CI = −48.195 to 19.315, *P* = .311).

When comparing women across age groups, no significant difference in clozapine concentrations was observed between women aged <45 and those aged 45-55 years (estimate = −9.902, CI = −38.635 to 18.830, *P* = .363), but women aged >55 years had significantly lower concentrations compared to those aged <45 years (estimate = −45.111, CI = −83.987 to −6.235, *P* = .004) and 45-55 years (estimate = −35.209, CI = −65.217 to −5.201, *P* = .004).

### Olanzapine

**Figure 3:**
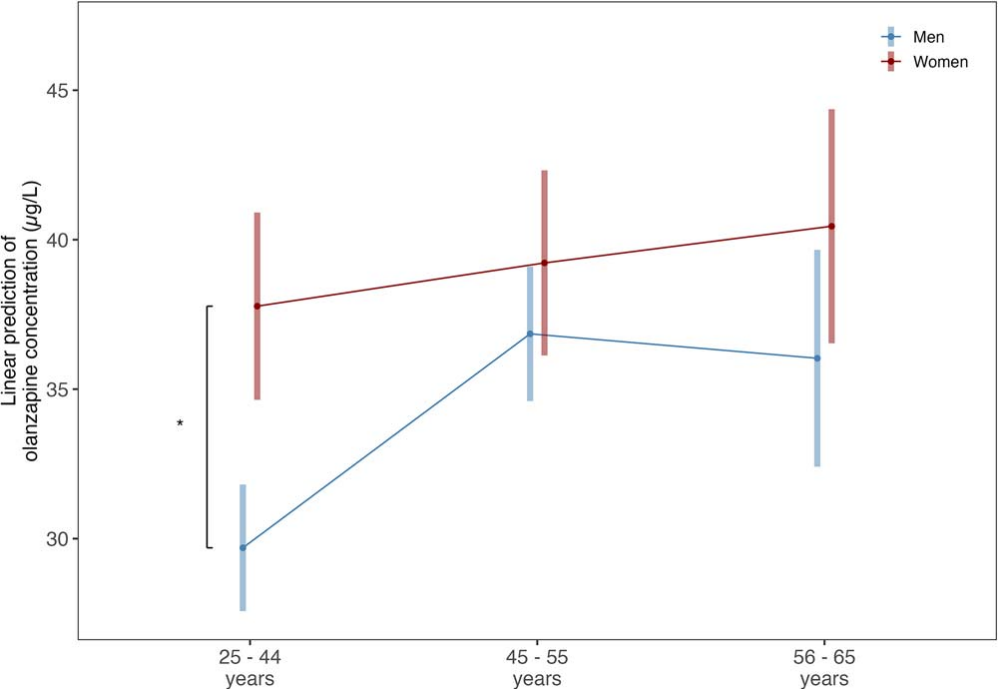
Estimated Marginalized Means (SE) for Olanzapine Serum Concentrations (μL) for Men (3740 samples of 1490 men) and Women (1374 samples of 751 women) in the Age Groups of <45, 45-55, and >55 years.

The sample consisted of 5114 unique samples of olanzapine serum concentrations from 2241 patients, of which 34% were female (*n* = 751). Linear mixed effect models showed that overall women had significantly higher olanzapine concentrations (patient-aggregated mean 38.45 μg/L, SD 26.64) than men (mean 32.35 μg/L, SD 21.73) (estimate = 8.08, CI = 4.93 to 11.23, *P* < .001, Figure 3).

Young women (<45 years) had significantly higher olanzapine concentrations than men (estimate = 8.083, CI = 3.843 to 12.324, *P* < .001), but at ages 45-55 and over 55 years (estimate = 0.876, CI = −4.342 to 6.093, *P* = .658 for 45-55; estimate = 4.416, CI = −0.659 to 9.492, *P* = .065 for >55) no significant sex differences were present.

Among women, olanzapine concentrations did not differ between age groups (estimate = 1.447, CI = −4.840 to 7.733, *P* = .562 for <45 vs 45-55 years; estimate = 2.674, CI = −5.088 to 10.437, *P* = .545 for <45 vs >55 years; estimate = 1.227, CI = −4.353 to 6.808, *P* = .562 for 45-55 vs >55 years).

### Aripiprazole

**Figure 4:**
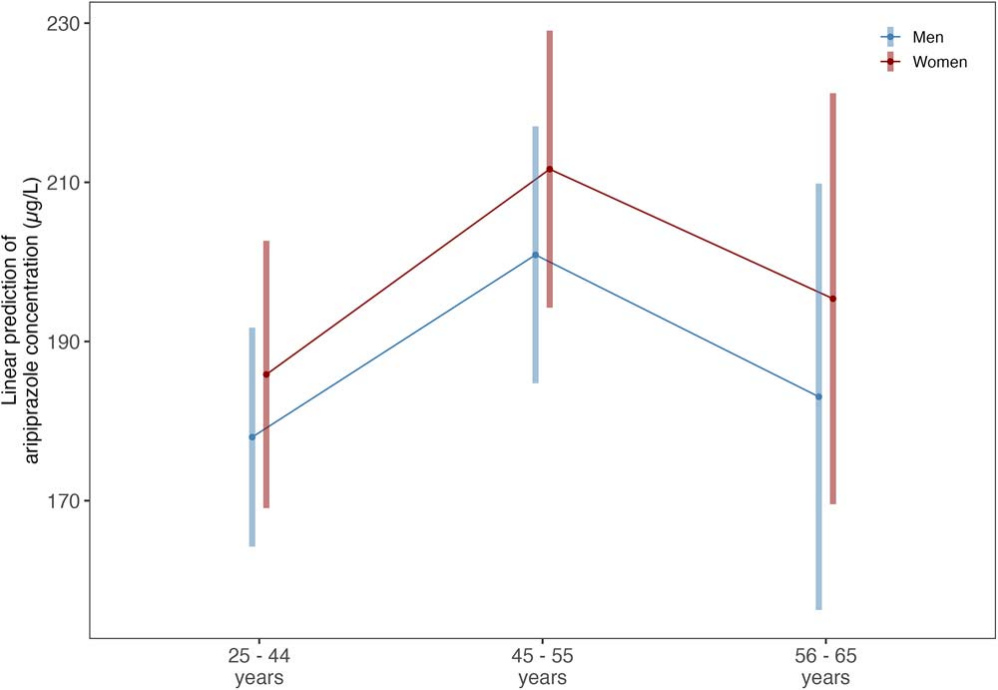
Estimated Marginalized Means (SE) Corrected for Age and Repeated Measurements for Aripiprazole Serum concentrations (μL) for men (2013 samples of 806 men) and women (1158 samples of 563 women) in the age groups of <45, 45-55, and >55 years.

A total of 3171 samples were present from 1369 patients of whom 41% (n = 563) were female. Overall, aripiprazole concentrations did not differ significantly between men and women (patient-aggregated mean 194.83, SD 135.31 for women; mean 181.87, SD 120.54 for men, estimate = 7.87, CI = −10.56 to 26.30, *P* = .402, Figure 4). Similarly, dose-corrected aripiprazole concentrations and prescribed doses were similar in both sexes (Appendix B).

Estimated marginalized means showed no significant differences within any age group (estimate = 7.872, CI = −16.929 to 32.673, *P* = .489 for <45 years; estimate = 10.767, CI = −19.084 to 40.619, *P* = .489 for 45-55 years; estimate = 12.319, CI = −26.907 to 51.545, *P* = .489 for >55 years).

Among women, concentrations were similar across all age groups (estimate = 25.794, CI = −7.212 to 58.799, *P* = .235 for <45 vs 45-55; estimate = 9.521, CI = −36.880 to 55.922, *P* = .588 for <45 vs >55; estimate = −16.272, CI = −50.919 to 18.375, *P* = .489 for 45-55 vs >55 years).

### Quetiapine

**Figure 5:**
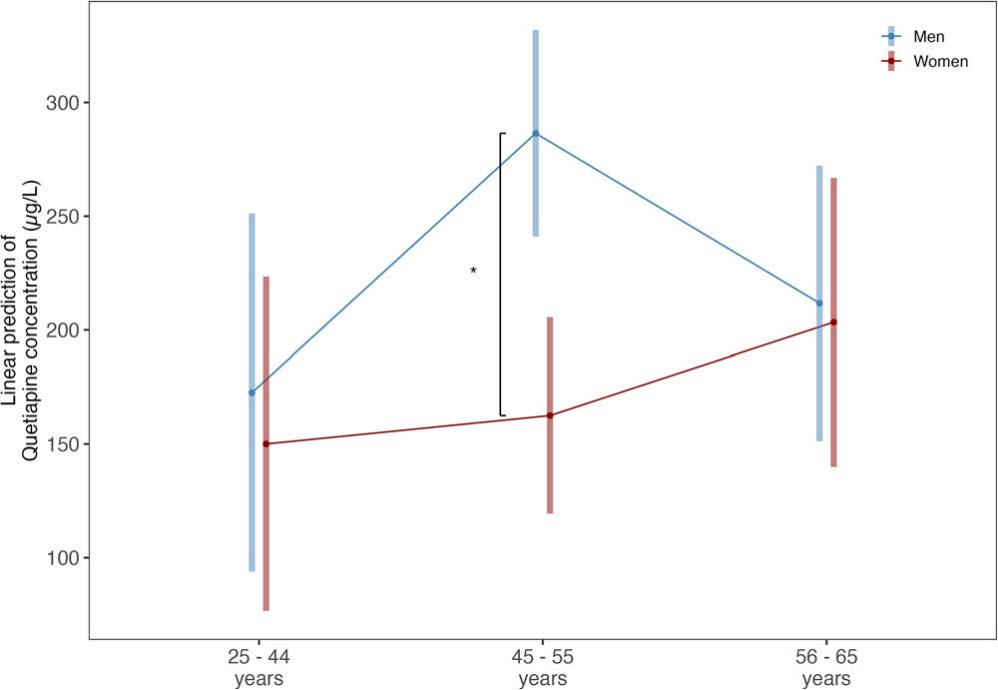
Estimated Marginalized Means (SE) Corrected for Age and Repeated Measurements for Quetiapine Serum Concentrations (μL) for Men (667 Samples of 335 Men) and Women (665 samples of 396 women) in the Age Groups of <45, 45-55, and >55 years.

A total of 1332 quetiapine samples were analyzed from 731 patients of whom 54% (*n* = 396) were female. Mean concentrations were similar between women and men (patient-aggregated mean 175.4, SD 238.65 for women; mean 208.48, SD 316.32 for men; estimate = −22.51, CI = −99.06 to 54.04, *P* = .564, Figure 5), and no sex differences were found in C/D ratios and prescribed doses (Appendix B).

Comparing different age groups, quetiapine concentrations did not differ between men and women younger than 45 years (estimate = −22.510, CI = −125.784 to 80.764, *P* = .808) or over 55 years (estimate = −8.354, CI = −99.175 to 82.467, *P* = .808). However, women aged 45-55 years had significantly lower concentrations than men in the same age group (estimate = −123.939, CI = −208.451 to −39.427, *P* = .001).

Among women, quetiapine concentrations did not differ significantly between any of the age groups (estimate = 12.457, CI = −95.203 to 120.116, *P* = .808 for <45 vs 45-55 years; estimate = 53.345, CI = −104.813 to 211.502, *P* = .746 for <45 vs >55; estimate = 40.888, CI = −60.352 to 142.128, *P* = .746 for 45-55 vs >55).

## Discussion

In a sample of 44 378 blood measurements from 6147 patients, women had significantly higher serum concentrations than men for clozapine and olanzapine, but not for aripiprazole and quetiapine. When stratifying by age, olanzapine and clozapine concentrations were higher in women than in men before the age of 45, while no sex differences were found in older age groups. Conversely, we found higher levels of quetiapine in men as compared to women between 45 and 55 years. Among women, concentrations were similar across age groups for olanzapine, aripiprazole, and quetiapine, while women showed lower levels of clozapine after the age of 55. Overall, our findings for clozapine and olanzapine align with previous TDM trials showing most pronounced sex differences of these two drugs, whereas we did not replicate earlier reported sex differences for aripiprazole and quetiapine.^35^^,^^36^^,^^42^

The finding of higher concentrations of clozapine and olanzapine in younger women is in line with the key role for the CYP1A2 enzyme, which is inhibited by estrogen, leading to slower drug metabolism in premenopausal women. A proportion of these women likely used ethinylestradiol-containing contraceptives, leading to even higher plasma concentrations, as ethinylestradiol is an even stronger inhibitor of CYP1A2 and may require halving the dose of CYP1A2 substrates.^43^ The risk of overdosing younger women with clozapine and olanzapine is worrisome, not only due to the increased risk for side effects, but also because women may require lower instead of higher blood levels to achieve similar efficacy. Transporter molecules that pump medication out of the brain are inhibited by estrogen,^44^ resulting in higher drug concentrations in the brain. Women have 10%-15% greater blood flow to the brain, which makes it easier for a drug to reach their target receptor.^45^ Within the central nervous system, estrogens enhance dopamine D_2_ receptor sensitivity, potentially augmenting the effects of D_2_ receptor antagonists.^27^^,^^46^ Eugene and Misiak showed that premenopausal women require only half of the olanzapine dosage as compared to men to achieve equal occupancy of the dopamine D_2_ receptor,^46^ an effect that may be partly explained by the modulation of D_2_ receptors by estrogen.

During the menopausal transition, we expected lower clozapine and olanzapine concentrations in women due to increasing CYP1A2 activity in the absence of estrogen. Indeed, for clozapine, serum concentrations were lower in women over 55 years compared to women younger than 55 years. With declining clozapine concentrations and typically lower prescribed doses after age 50, both TDM and careful monitoring of symptoms and overall functioning are particularly important during the menopausal transition to ensure treatment is tailored to individual needs. Although sex differences for olanzapine decline with age, concentrations in women did not change significantly after age 55, either reflecting slower metabolism with age or the influence of a broader enzyme profile involved.^45^ Although we had incomplete information on olanzapine dosages, we did observe an increase in dosage among older women. Speculatively, given that concentrations are expected to decrease in the absence of estrogen, this could suggest that dosing adjustments for olanzapine may already be occurring, possibly guided by TDM and clinical evaluation.

We found no overall sex differences for quetiapine, in contrast to Jönsson who observed lower dose-corrected serum levels in men.^35^ We found higher quetiapine concentrations in men than in women between 45 and 55 years of age, which could reflect increased CYP3A4 activity during the menopausal transition, a period marked by substantial fluctuations in estrogen, potentially leading to a net increase in oestradiol.^47^ While our study is the first to examine sex differences stratified by age groups as a proxy for menopausal status, Castberg,^36^ also reported no sex differences at younger ages but noted increasing concentrations in women with advancing age. Although this does not directly align with our findings, it does support the hypothesis that estradiol may induce quetiapine metabolism, and that this may increase concentrations after menopause once estrogen has declined.

We did not observe any sex differences in aripiprazole concentrations, which contrasts with the findings reported by Jönsson et al.^35^ A key limitation of our analysis is the incomplete dosing information for aripiprazole, raising the possibility that observed similarities in plasma concentrations may reflect lower prescribed doses in women as compared to men. Aripiprazole is primarily metabolized by CYP2D6, an enzyme not significantly influenced by estrogen, though sex-related differences in absorption, distribution, or elimination are still expected to yield higher concentrations in women. However, aripiprazole is also partially metabolized by CYP3A4, which is estrogen-induced and may counterbalance other sex-related pharmacokinetic differences in a similar way as for quetiapine.

Overall, our findings highlight the need for systematic investigation into pharmacokinetic sex differences in antipsychotic response and underscore that the influence of sex is neither uniform across different antipsychotics, nor constant across different life phases. Women have historically been excluded from clinical and pharmaceutical trials, mainly to avoid the potential risk that comes with the inclusion of individuals with childbearing potential, and to avoid the variability that is said to come with their menstrual cycle.^48-50^ Although the proportion of women in clinical trials has increased in the past few decades, sex-specific analyses remain the exception rather than the rule.^49^ As a result, the doses of most drugs used today are prescribed using a one-size-fits-all approach, while being based on male-dominated clinical trials. At the same time, sex differences in pharmacokinetics predict the occurrence of side effects in women, but not in men,^15^ and adverse events databases show that women experience more adverse events than men.^15^^,^^16^^,^^51-53^ The current study reinforces the critical need for sex-stratified analyses in clinical trials investigating pharmacological strategies, and suggest that sex- and age-informed prescribing strategies are essential to optimize both efficacy and tolerability of antipsychotic treatment in the case of clozapine and olanzapine.

The main limitation of the study is the incomplete data on dose-adjusted concentrations for olanzapine, aripiprazole, and quetiapine. In addition, we were unable to assess the exact impact of menopause on antipsychotic efficacy and tolerability, as the date of menopause was unavailable and menopausal status was approximated using age. Furthermore, the analyses could not be corrected or stratified for smoking status, diagnosis, medical comorbidities, reasons for blood sampling, validated menopausal status, drug compliance, exogenous hormone use (eg, hormonal contraceptives or hormone replacement therapy), and concomitant use of medications with hepatic impacts other than psychotropics, yet the large samples of the study limit their impact. Smoking status is more prevalent among men with psychotic disorders and has been shown to significantly reduce clozapine levels by increasing CYP1A2 activity,^54-57^ while oral contraceptive use has been related to a two- to threefold increase in clozapine levels, and may potentially also influence olanzapine metabolism in a similar manner.^58^ Future studies should also assess norclozapine, as the norclozapine-to-clozapine ratio provides an important indicator of CYP1A2 activity and could offer further insight into potential sex differences in metabolism and treatment response.

## Conclusion

In this large TDM sample, we found pronounced sex differences in antipsychotic concentrations, which varied by compound and across age groups. Women showed significantly higher serum concentrations of clozapine and olanzapine compared with men only before the age of 45. In contrast, quetiapine concentrations were lower in women than in men during the perimenopausal period, potentially reflecting increased estrogen-related CYP3A4 activity, while no sex differences were observed for aripiprazole, consistent with its CYP2D6 and CYP3A4-driven metabolism. Taken together, these results highlight the need for sex- and age-sensitive approaches in antipsychotic prescribing. Systematic inclusion of sex and female life phase as biological variables, alongside stratification by age, is essential to move towards personalized prescription of antipsychotic medication.

## Supplementary Material

Appendix_Antipsychotic_concentrations_sex_and_menopausal_age_sbaf217
