# Supplementary material for: The Impact of Sex and Age on Antipsychotic Serum Concentrations
Source: Schizophr Bull. 2026 Mar 21;52(2):sbaf217. doi: 10.1093/schbul/sbaf217 (PMC13005119; doi:10.1093/schbul/sbaf217)
Supplement: Appendix_Antipsychotic_concentrations_sex_and_menopausal_age_sbaf217 [file appendix_antipsychotic_concentrations_sex_and_menopausal_age_sbaf217.docx]

Appendix A. Linear mixed effects models (LMEM) and contrasts of marginalized means

**1. Clozapine**

**Table A1.** Clozapine LMEM results

| ***Predictors*** | ***Estimates*** | ***95% CI*** | ***p-value*** |
| --- | --- | --- | --- |
| Intercept | 274.85 | 244.06 – 305.64 | **<0.001** |
| Age (weeks) | 0.03 | 0.02 – 0.05 | **<0.001** |
| Sex: Female | 32.90 | 12.49 – 53.30 | **0.002** |
| Age group: 45-55 years | 6.34 | -4.99 – 17.66 | 0.273 |
| Age group: >55 years | 2.23 | -15.97 – 20.42 | 0.810 |
| Sex: Female × Age group: 45-55 years | -16.24 | -38.62 – 6.15 | 0.155 |
| Sex: Female × Age group: >55 years | -47.34 | -76.95 – -17.72 | **0.002** |

**Table A2.** Contrasts of estimated marginalized means

| **contrast** | **estimate** | **z-ratio** | **p-value** | **95% CI lower limit** | **95% CI upper limit** |
| --- | --- | --- | --- | --- | --- |
| Men vs. women <45 years | 32.898 | 3.160 | 0.004 | 5.432 | 60.364 |
| Men vs. women 45-55 years | 16.661 | 1.604 | 0.163 | -10.748 | 44.069 |
| Men vs. women >55 years | -14.440 | -1.129 | 0.311 | -48.195 | 19.315 |
| Women <45 vs. women 45-55 years | -9.902 | -0.909 | 0.363 | -38.635 | 18.830 |
| Women <45 vs. women >55 years | -45.111 | -3.061 | 0.004 | -83.987 | -6.235 |
| Women 45-55 vs. women >55 years | -35.209 | -3.096 | 0.004 | -65.217 | -5.201 |

**2. Olanzapine**

**Table A3.** Olanzapine LMEM results

| ***Predictors*** | ***Estimates*** | ***95% CI*** | ***p-value*** |
| --- | --- | --- | --- |
| Intercept | 34.27 | 27.93 – 40.62 | **<0.001** |
| Age (weeks) | -0.00 | -0.01 – 0.00 | 0.242 |
| Sex: Female | 8.08 | 4.93 – 11.23 | **<0.001** |
| Age group: 45-55 years | 7.16 | 3.83 – 10.49 | **<0.001** |
| Age group: >55 years | 6.34 | 1.33 – 11.36 | **0.013** |
| Sex: Female × Age group: 45-55 years | -5.71 | -10.31 – -1.11 | **0.015** |
| Sex: Female × Age group: >55 years | -3.67 | -8.56 – 1.22 | 0.142 |

**Table A4.** Contrasts of estimated marginalized means

| **contrast** | **estimate** | **z-ratio** | **p-value** | **95% CI lower limit** | **95% CI upper limit** |
| --- | --- | --- | --- | --- | --- |
| Men vs. women <45 years | 8.083 | 5.029 | 0.000 | 3.843 | 12.324 |
| Men vs. women 45-55 years | 2.372 | 1.294 | 0.391 | -2.463 | 7.208 |
| Men vs. women >55 years | 4.416 | 2.296 | 0.065 | -0.659 | 9.492 |
| Women <45 vs. women 45-55 years | 1.447 | 0.607 | 0.562 | -4.840 | 7.733 |
| Women <45 vs. women >55 years | 2.674 | 0.909 | 0.545 | -5.088 | 10.437 |
| Women 45-55 vs. women >55 years | 1.227 | 0.580 | 0.562 | -4.353 | 6.808 |

**3. Aripirazole**

**Table A5.** Aripiprazole LMEM results

| ***Predictors*** | ***Estimates*** | ***95% CI*** | ***p-value*** |
| --- | --- | --- | --- |
| Intercept | 165.00 | 123.02 – 206.97 | **<0.001** |
| Age (weeks) | 0.01 | -0.02 – 0.03 | 0.606 |
| Sex: Female | 7.87 | -10.56 – 26.30 | 0.402 |
| Age group: 45-55 years | 22.90 | 0.27 – 45.52 | **0.047** |
| Age group: >55 years | 5.07 | -29.15 – 39.30 | 0.771 |
| Sex: Female × Age group: 45-55 years | 2.90 | -23.84 – 29.63 | 0.832 |
| Sex: Female × Age group: >55 years | 4.45 | -29.60 – 38.49 | 0.798 |

**Table A6**. Contrasts of estimated marginalized means

| **contrast** | **estimate** | **z-ratio** | **p-value** | **95% CI lower limit** | **95% CI upper limit** |
| --- | --- | --- | --- | --- | --- |
| Men vs. women <45 years | 7.872 | 0.837 | 0.489 | -16.929 | 32.673 |
| Men vs. women 45-55 years | 10.767 | 0.952 | 0.489 | -19.084 | 40.619 |
| Men vs. women >55 years | 12.319 | 0.829 | 0.489 | -26.907 | 51.545 |
| Women <45 vs. women 45-55 years | 25.794 | 2.062 | 0.235 | -7.212 | 58.799 |
| Women <45 vs. women >55 years | 9.521 | 0.541 | 0.588 | -36.880 | 55.922 |
| Women 45-55 vs. women >55 years | -16.272 | -1.239 | 0.489 | -50.919 | 18.375 |

**4. Quetiapine**

**Table A7***.* Quetiapine LMEM results

| ***Predictors*** | ***Estimates*** | ***95% CI*** | ***P-value*** |
| --- | --- | --- | --- |
| Intercept | 138.18 | -0.56 – 276.93 | 0.051 |
| Age (weeks) | 0.01 | -0.06 – 0.08 | 0.824 |
| Sex: Female | 12.16 | -46.57 – 70.89 | 0.685 |
| Age group: 45-55 years | 125.23 | 44.58 – 205.89 | **0.002** |
| Age group: >55 years | 46.68 | -53.91 – 147.28 | 0.363 |
| Sex: Female × Age group: 45-55 years | -132.31 | -213.72 – -50.91 | **0.001** |
| Sex: Female × Age group: >55 years | -21.90 | -108.91 – 65.11 | 0.622 |

**Table A8.** Contrasts of estimated marginalized means

| **contrast** | **estimate** | **t-ratio** | **p-value** | **95% CI lower limit** | **95% CI upper limit** |
| --- | --- | --- | --- | --- | --- |
| Men vs. women <45 years | -22.510 | -0.577 | 0.808 | -125.784 | 80.764 |
| Men vs. women 45-55 years | -123.939 | -3.878 | 0.001 | -208.451 | -39.427 |
| Men vs. women >55 years | -8.354 | -0.243 | 0.808 | -99.175 | 82.467 |
| Women <45 vs. women 45-55 years | 12.457 | 0.306 | 0.808 | -95.203 | 120.116 |
| Women <45 vs. women >55 years | 53.345 | 0.891 | 0.746 | -104.813 | 211.502 |
| Women 45-55 vs. women >55 years | 40.888 | 1.067 | 0.746 | -60.352 | 142.128 |

Appendix B. Antipsychotic C/D ratios and prescribed doses

**1. Clozapine**

**Table A9.** Clozapine C/D ratio by sex and age group

| Group | Individuals (n, %) | Measurements (n, %) | Mean | Median | SD | IQR |
| --- | --- | --- | --- | --- | --- | --- |
| All | 628 (100%) | 3973 (100%) | 1.64 | 1.19 | 2.16 | 1.12 |
| Men | 457 (72.8%) | 2959 (74.5%) | 1.54 | 1.10 | 2.35 | 0.99 |
| Women | 171 (27.2%) | 1014 (25.5%) | 1.92 | 1.57 | 1.42 | 1.33 |
| Men <45 years | 158 (25.2%) | 962 (24.2%) | 1.78 | 1.07 | 3.60 | 0.96 |
| Women <45 years | 43 (6.8%) | 250 (6.3%) | 1.90 | 1.69 | 1.62 | 1.35 |
| Men 45-55 years | 247 (39.3%) | 1508 (38%) | 1.41 | 1.09 | 1.45 | 0.98 |
| Women 45-55 years | 97 (15.4%) | 537 (13.5%) | 1.88 | 1.52 | 1.28 | 1.43 |
| Men >55 years | 89 (14.2%) | 489 (12.3%) | 1.47 | 1.20 | 1.09 | 1.08 |

| 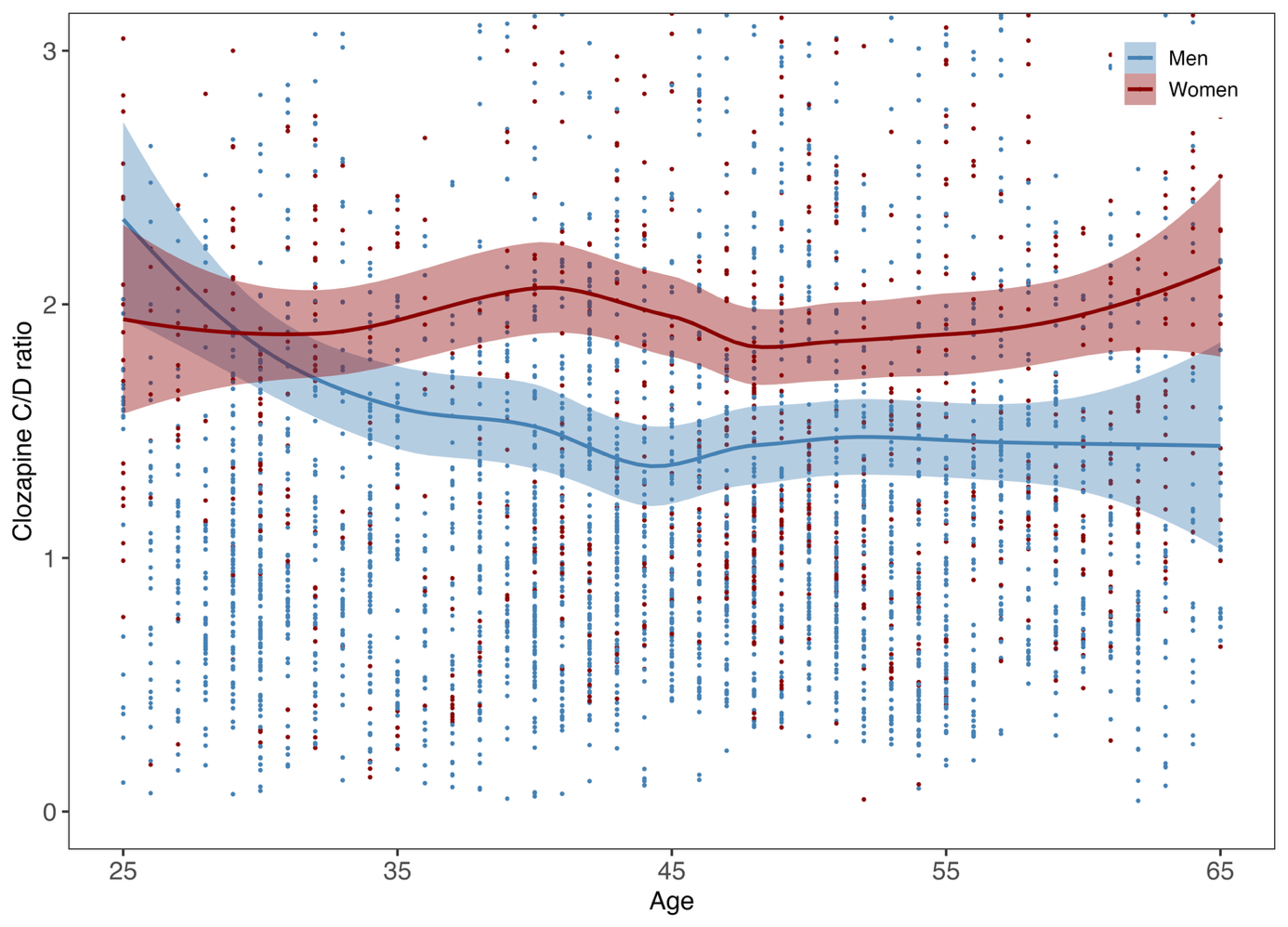 |
| --- |
| **Figure A1.** Clozapine C/D ratios for men and women across ages |

**Table A0***.* Clozapine prescribed dose (mg/day) by sex and age group

| Group | Individuals (n, %) | Measurements (n, %) | Mean | Median | SD | IQR |
| --- | --- | --- | --- | --- | --- | --- |
| All | 628 (100%) | 7930 (100%) | 338.17 | 300 | 172.60 | 250 |
| Men | 457 (72.8%) | 5906 (74.5%) | 356.05 | 350 | 176.14 | 275 |
| Women | 171 (27.2%) | 2024 (25.5%) | 286.01 | 275 | 150.15 | 150 |
| Men <45 years | 158 (25.2%) | 1921 (24.2%) | 335.98 | 300 | 174.99 | 225 |
| Women <45 years | 43 (6.8%) | 499 (6.3%) | 332.72 | 300 | 197.17 | 200 |
| Men 45-55 years | 247 (39.3%) | 3009 (37.9%) | 370.02 | 350 | 178.42 | 275 |
| Women 45-55 years | 97 (15.4%) | 1073 (13.5%) | 283.74 | 300 | 135.39 | 175 |
| Men >55 years | 89 (14.2%) | 976 (12.3%) | 352.46 | 350 | 167.31 | 250 |
| Women >55 years | 42 (6.7%) | 452 (5.7%) | 239.82 | 250 | 100.33 | 150 |

| 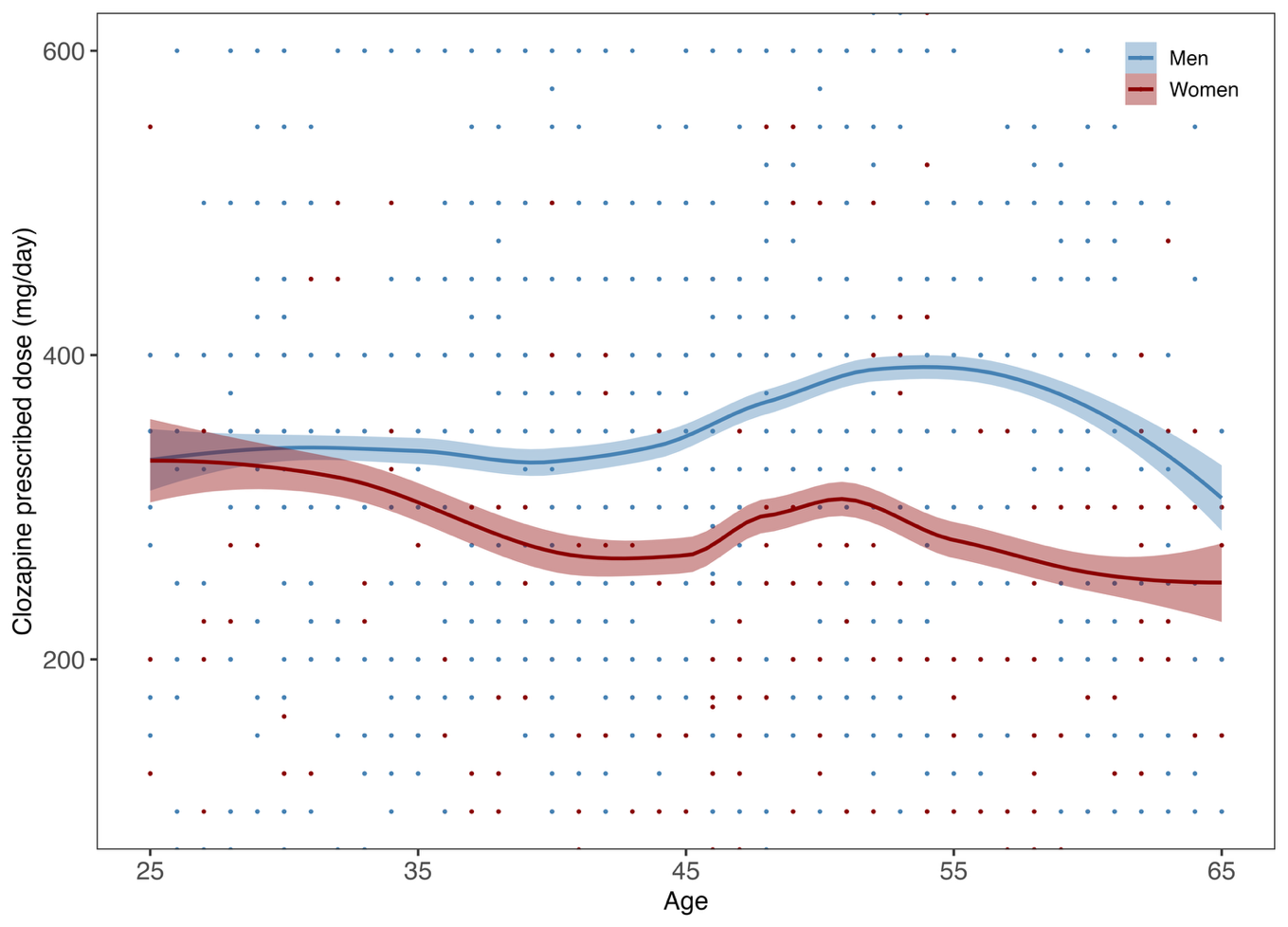 |
| --- |
| **Figure A2.** Clozapine prescribed dose (mg/day) for men and women across ages |

**2. Olanzapine**

**Table A11.** Olanzapine C/D ratio by sex and age group

| Group | Individuals (n, %) | Measurements (n, %) | Mean | Median | SD | IQR |
| --- | --- | --- | --- | --- | --- | --- |
| All | 109 (100%) | 205 (100%) | 2.95 | 2.23 | 3.02 | 1.67 |
| Men | 74 (67.9%) | 145 (70.7%) | 2.85 | 2.20 | 3.24 | 1.55 |
| Women | 35 (32.1%) | 60 (29.3%) | 3.20 | 2.40 | 2.39 | 2.03 |
| Men <45 years | 25 (22.9%) | 57 (27.8%) | 3.20 | 2.20 | 3.53 | 1.80 |
| Women <45 years | 9 (8.3%) | 9 (4.4%) | 2.96 | 2.20 | 1.85 | 2.08 |
| Men 45-55 years | 30 (27.5%) | 50 (24.4%) | 2.95 | 2.28 | 3.95 | 1.65 |
| Women 45-55 years | 12 (11%) | 19 (9.3%) | 3.58 | 2.27 | 3.61 | 2.38 |
| Men >55 years | 22 (20.2%) | 38 (18.5%) | 2.21 | 2.13 | 0.92 | 1.15 |
| Women >55 years | 15 (13.8%) | 32 (15.6%) | 3.04 | 2.48 | 1.50 | 1.62 |

| 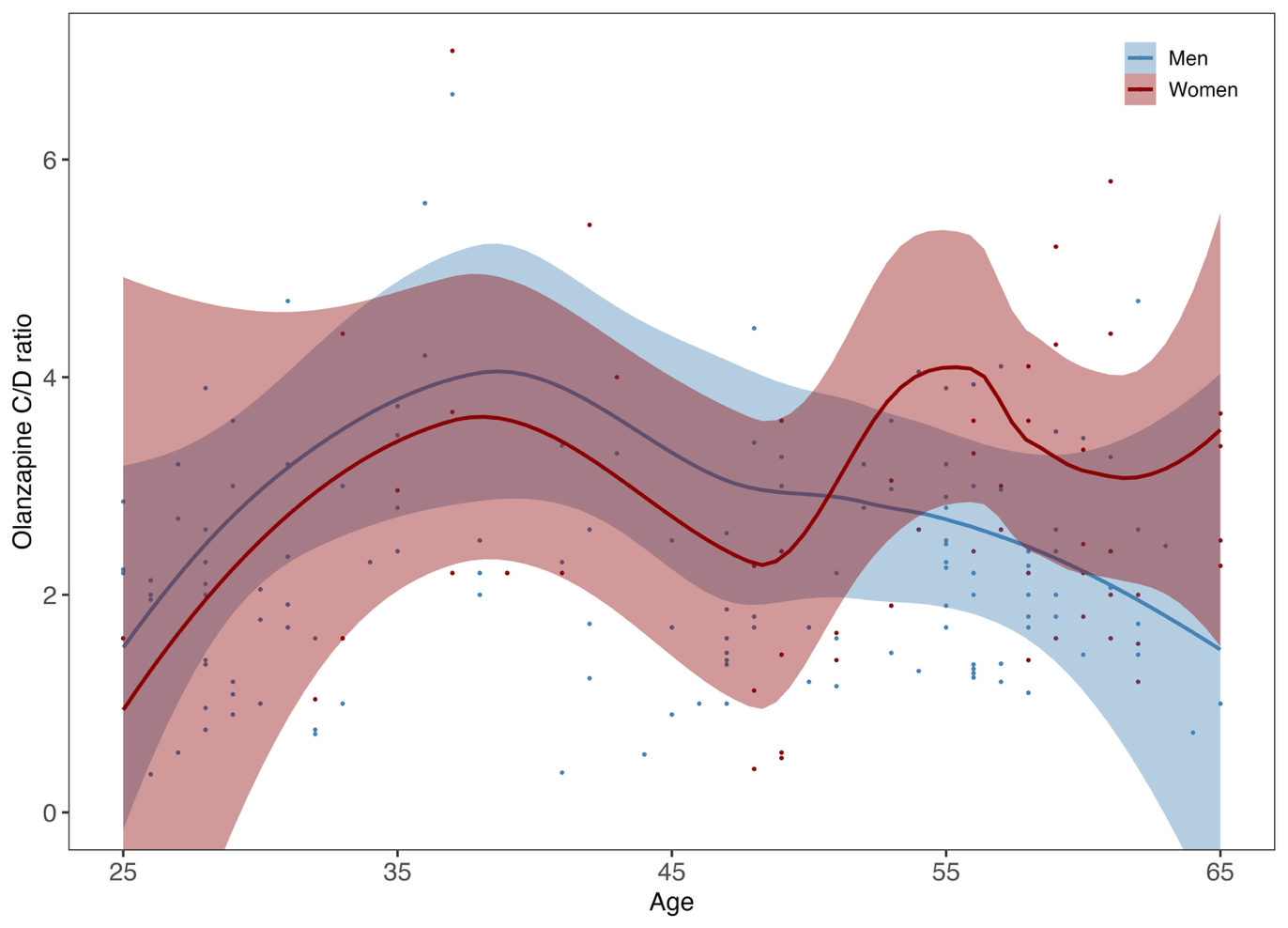 |
| --- |
| **Figure A3.** Olanzapine C/D ratios for men and women across ages |

**Table A12.** Olanzapine prescribed dose (mg/day) by sex and age group

| Group | Individuals (n, %) | Measurements (n, %) | Mean | Median | SD | IQR |
| --- | --- | --- | --- | --- | --- | --- |
| All | 109 (100%) | 205 (100%) | 14.51 | 15.0 | 7.67 | 10.00 |
| Men | 74 (67.9%) | 145 (70.7%) | 15.17 | 15.0 | 7.63 | 10.00 |
| Women | 35 (32.1%) | 60 (29.3%) | 12.92 | 10.0 | 7.59 | 15.00 |
| Men <45 years | 25 (22.9%) | 57 (27.8%) | 13.49 | 10.0 | 7.29 | 12.50 |
| Women <45 years | 9 (8.3%) | 9 (4.4%) | 12.22 | 10.0 | 7.65 | 5.00 |
| Men 45-55 years | 30 (27.5%) | 50 (24.4%) | 14.90 | 12.5 | 7.84 | 10.00 |
| Women 45-55 years | 12 (11%) | 19 (9.3%) | 12.76 | 10.0 | 6.97 | 13.75 |
| Men >55 years | 22 (20.2%) | 38 (18.5%) | 18.03 | 20.0 | 7.22 | 10.00 |
| Women >55 years | 15 (13.8%) | 32 (15.6%) | 13.20 | 10.0 | 8.14 | 15.00 |

| 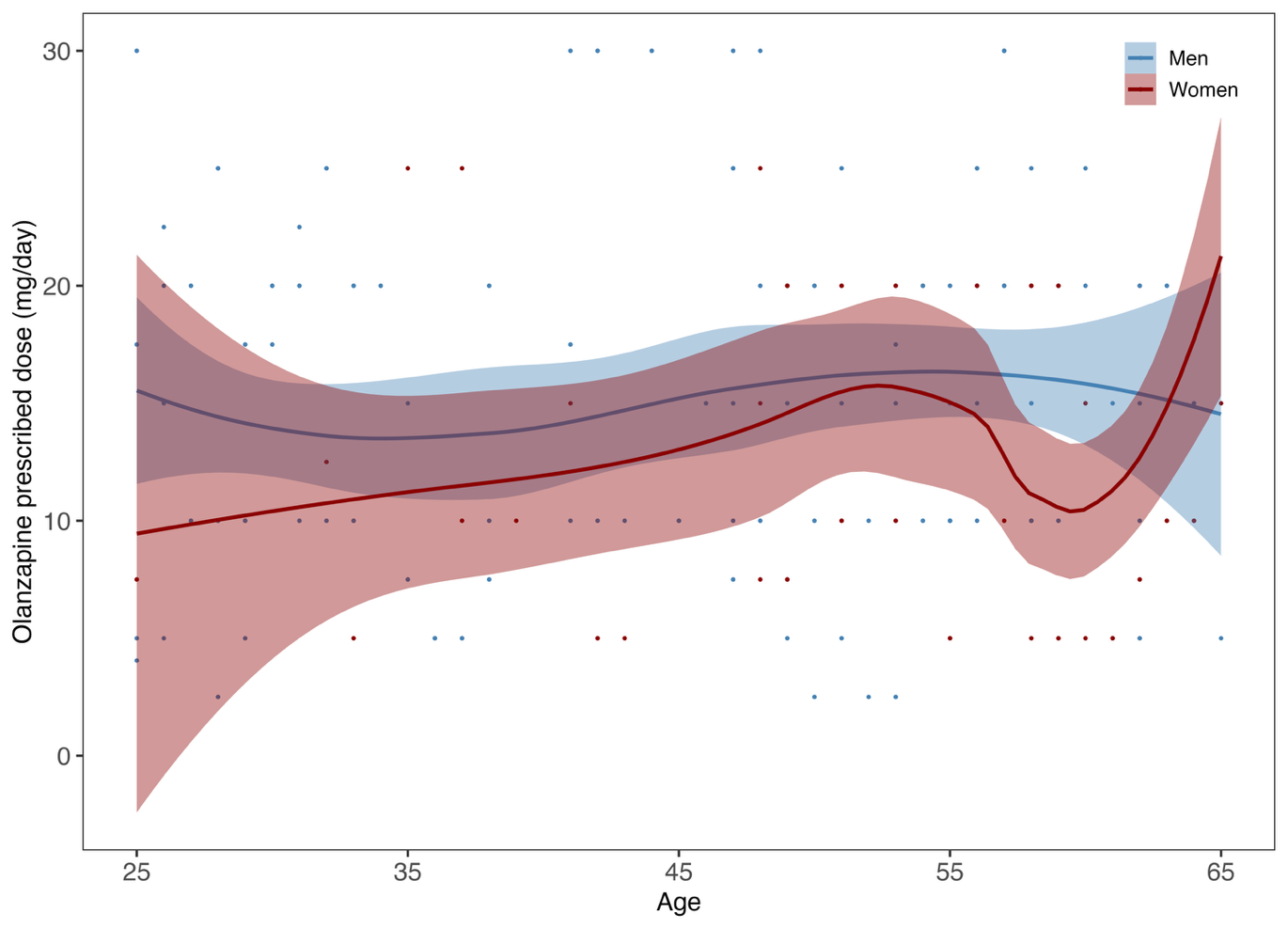 |
| --- |
| **Figure A4.** Olanzapine prescribed dose (mg/day) for men and women across ages |

**3. Aripiprazol**

**Table A13.** Aripiprazol C/D ratio by sex and age group

| Group | Individuals (n, %) | Measurements (n, %) | Mean | Median | SD | IQR |
| --- | --- | --- | --- | --- | --- | --- |
| All | 108 (100%) | 192 (100%) | 17.39 | 14.93 | 10.99 | 11.70 |
| Men | 63 (58.3%) | 119 (62%) | 17.82 | 15.00 | 11.90 | 14.15 |
| Women | 45 (41.7%) | 73 (38%) | 16.70 | 14.40 | 9.35 | 8.70 |
| Men <45 years | 25 (23.1%) | 39 (20.3%) | 20.47 | 17.20 | 13.69 | 19.32 |
| Women <45 years | 4 (3.7%) | 5 (2.6%) | 22.33 | 13.25 | 21.53 | 7.60 |
| Men 45-55 years | 32 (29.6%) | 63 (32.8%) | 16.41 | 13.50 | 11.71 | 10.63 |
| Women 45-55 years | 29 (26.9%) | 50 (26%) | 15.73 | 13.86 | 8.65 | 9.51 |
| Men >55 years | 10 (9.3%) | 17 (8.9%) | 16.96 | 15.33 | 6.58 | 10.07 |
| Women >55 years | 13 (12%) | 18 (9.4%) | 17.81 | 16.33 | 5.68 | 7.12 |

| 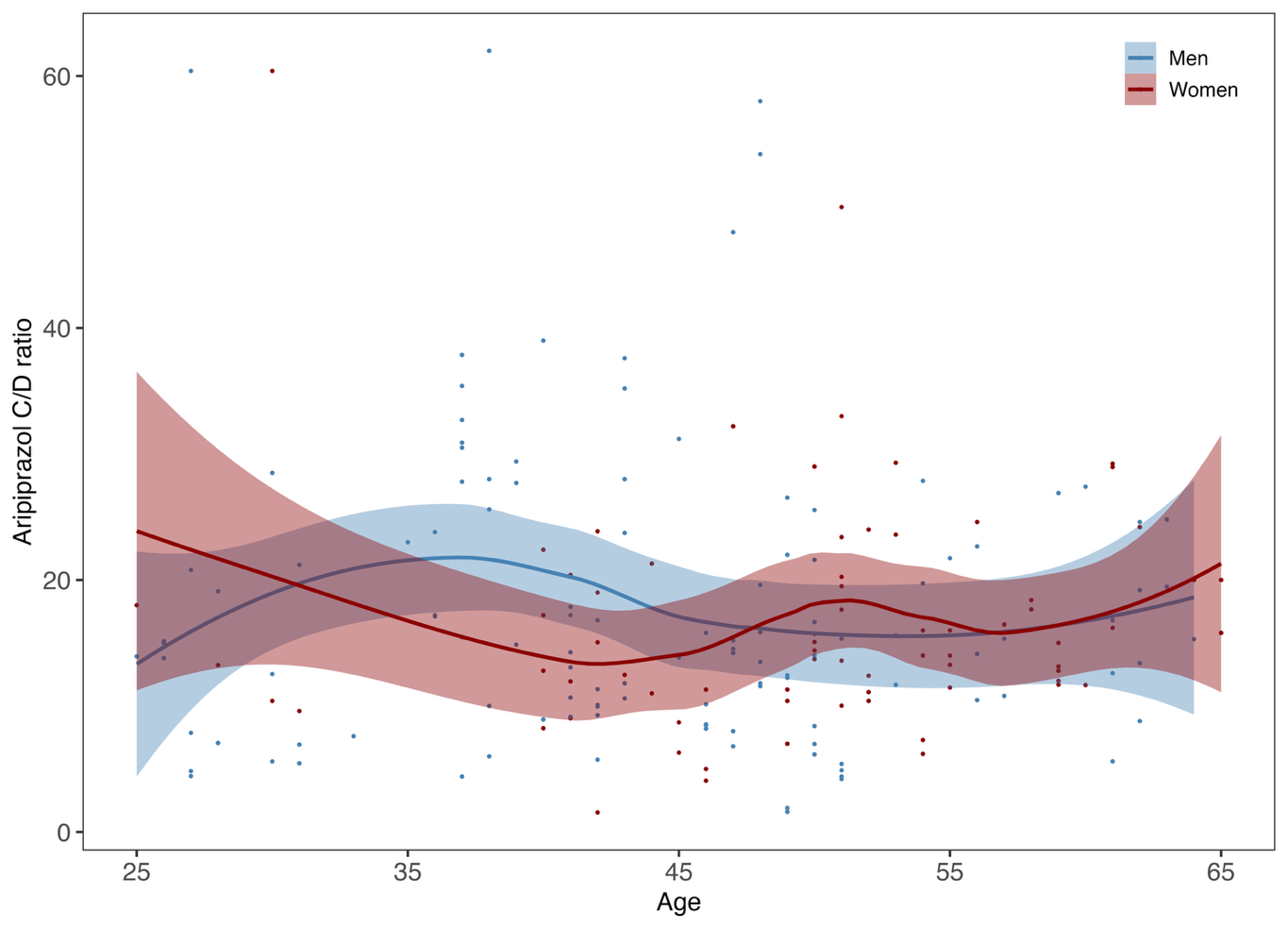 |
| --- |
| **Figure A5.** Aripiprazol C/D ratios for men and women across ages |

**Table A14.** Aripiprazol prescribed dose (mg/day) by sex and age group

| Group | Individuals (n, %) | Measurements (n, %) | Mean | Median | SD | IQR |
| --- | --- | --- | --- | --- | --- | --- |
| All | 108 (100%) | 192 (100%) | 14.02 | 10.0 | 9.32 | 12.5 |
| Men | 63 (58.3%) | 119 (62%) | 13.27 | 10.0 | 9.63 | 12.5 |
| Women | 45 (41.7%) | 73 (38%) | 15.24 | 15.0 | 8.72 | 10.0 |
| Men <45 years | 25 (23.1%) | 39 (20.3%) | 11.92 | 10.0 | 8.16 | 10.0 |
| Women <45 years | 4 (3.7%) | 5 (2.6%) | 9.50 | 7.5 | 6.47 | 2.5 |
| Men 45-55 years | 32 (29.6%) | 63 (32.8%) | 14.98 | 15.0 | 10.51 | 12.5 |
| Women 45-55 years | 29 (26.9%) | 50 (26%) | 15.70 | 15.0 | 9.19 | 10.0 |
| Men >55 years | 10 (9.3%) | 17 (8.9%) | 10.00 | 7.5 | 8.43 | 5.0 |
| Women >55 years | 13 (12%) | 18 (9.4%) | 15.56 | 15.0 | 7.65 | 5.0 |

| 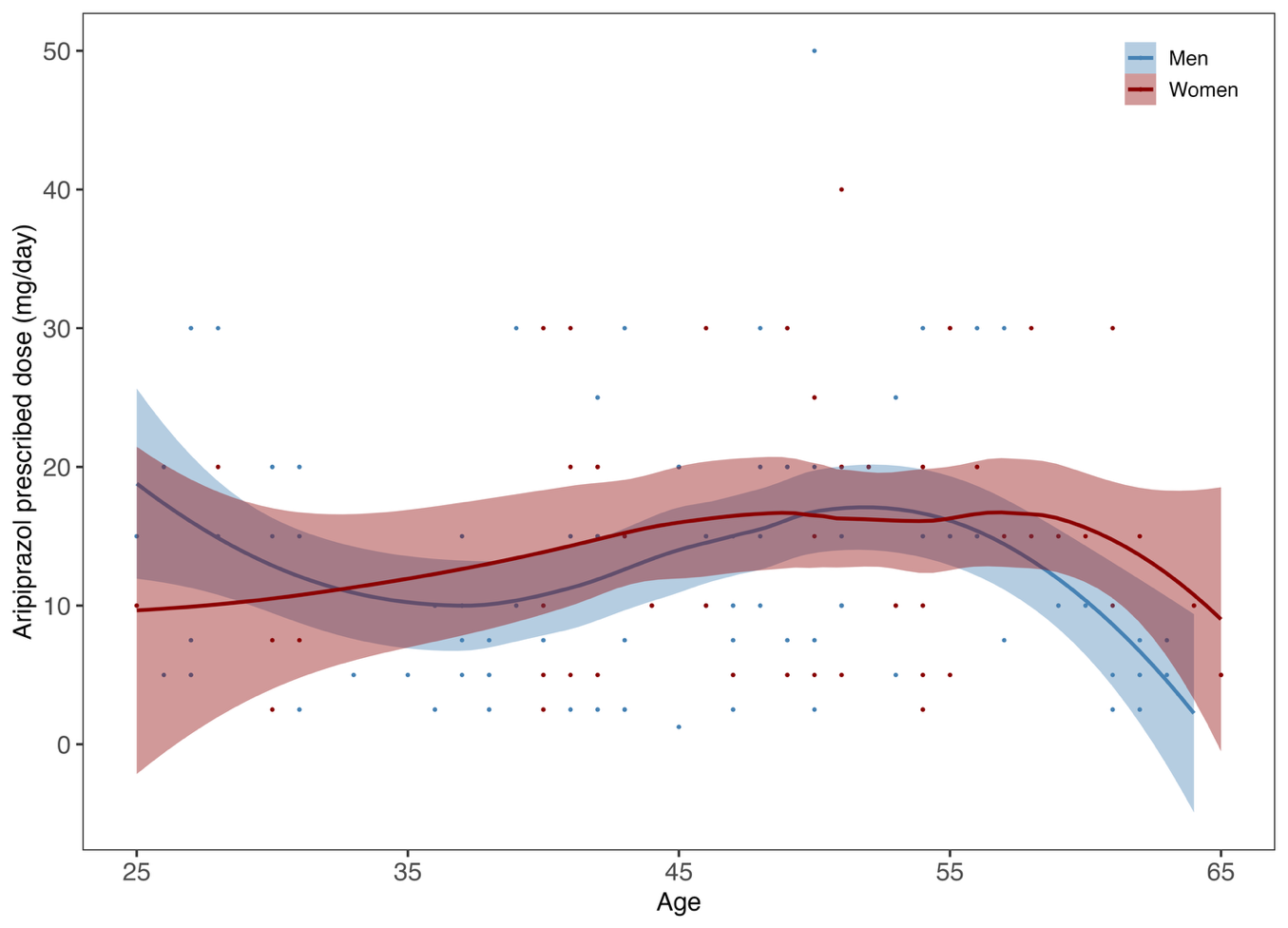 |
| --- |
| **Figure A6***.* Aripiprazol prescribed dose (mg/day) for men and women across ages |

**4. Quetiapine**

**Table A15.** Quetiapine C/D ratio by sex and age group

| Group | Individuals (n, %) | Measurements (n, %) | Mean | Median | SD | IQR |
| --- | --- | --- | --- | --- | --- | --- |
| All | 50 (100%) | 68 (100%) | 0.77 | 0.45 | 0.92 | 0.77 |
| Men | 14 (28%) | 19 (27.9%) | 0.88 | 0.43 | 1.12 | 0.73 |
| Women | 36 (72%) | 49 (72.1%) | 0.73 | 0.47 | 0.83 | 0.75 |
| Men <45 years | 3 (6%) | 4 (5.9%) | 1.87 | 1.29 | 1.71 | 1.66 |
| Women <45 years | 4 (8%) | 4 (5.9%) | 0.79 | 0.70 | 0.71 | 0.98 |
| Men 45-55 years | 7 (14%) | 9 (13.2%) | 0.55 | 0.35 | 0.61 | 0.29 |
| Women 45-55 years | 23 (46%) | 34 (50%) | 0.83 | 0.52 | 0.95 | 0.97 |
| Men >55 years | 4 (8%) | 6 (8.8%) | 0.72 | 0.15 | 1.06 | 0.72 |
| Women >55 years | 9 (18%) | 11 (16.2%) | 0.38 | 0.30 | 0.23 | 0.30 |

| 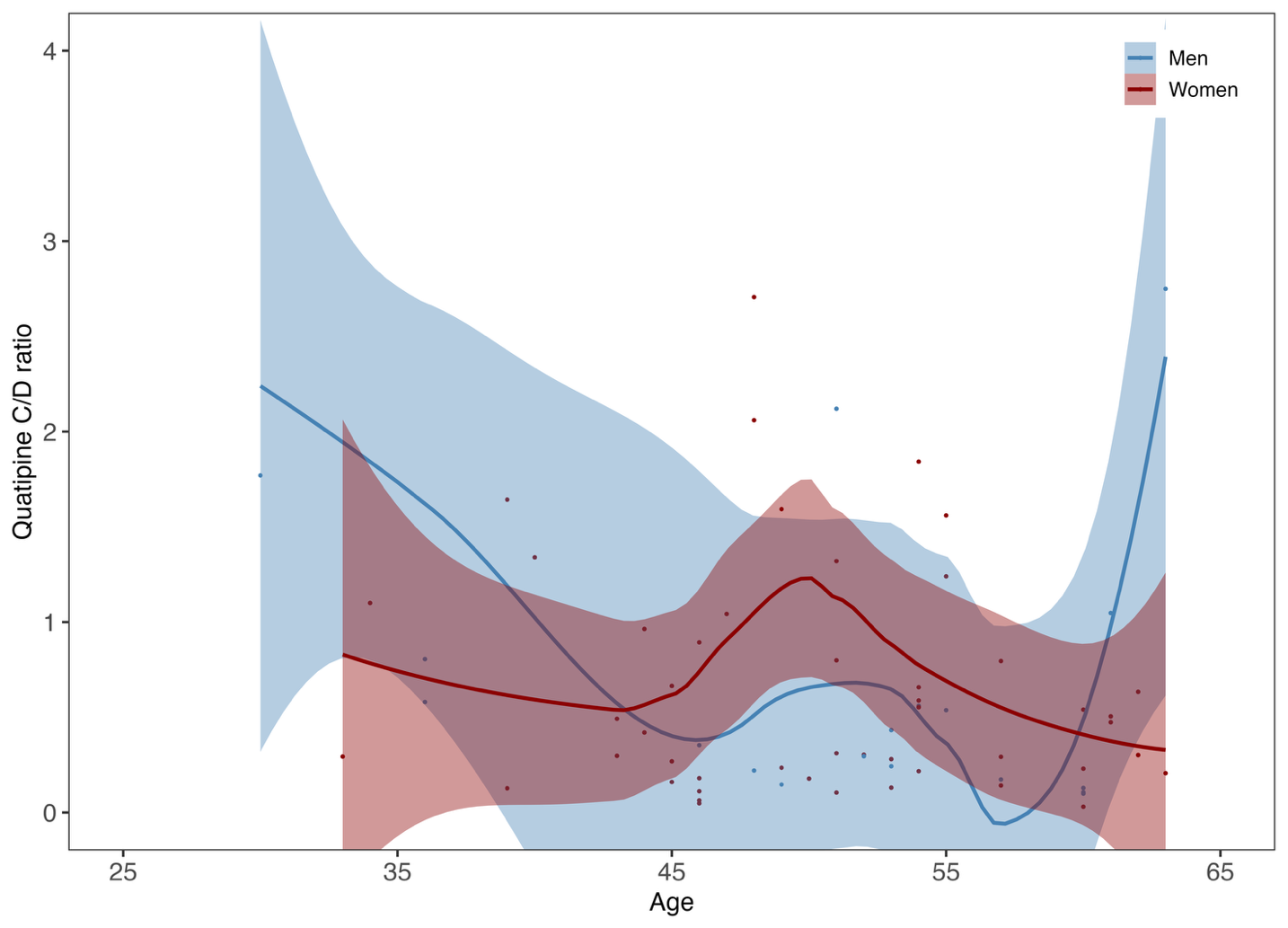 |
| --- |
| **Figure A7.** Quetiapine C/D ratios for men and women across ages |

**Table A16.** Quetiapine prescribed dose (mg/day) by sex and age group

| Group | Individuals (n, %) | Measurements (n, %) | Mean | Median | SD | IQR |
| --- | --- | --- | --- | --- | --- | --- |
| All | 50 (100%) | 68 (100%) | 373.64 | 350.00 | 252.53 | 428.75 |
| Men | 14 (28%) | 19 (27.9%) | 330.79 | 200.00 | 303.54 | 425.00 |
| Women | 36 (72%) | 49 (72.1%) | 390.26 | 400.00 | 231.20 | 350.00 |
| Men <45 years | 3 (6%) | 4 (5.9%) | 137.50 | 150.00 | 75.00 | 112.50 |
| Women <45 years | 4 (8%) | 4 (5.9%) | 261.88 | 218.75 | 287.05 | 419.38 |
| Men 45-55 years | 7 (14%) | 9 (13.2%) | 283.33 | 175.00 | 265.46 | 350.00 |
| Women 45-55 years | 23 (46%) | 34 (50%) | 365.44 | 375.00 | 193.75 | 237.50 |
| Men >55 years | 4 (8%) | 6 (8.8%) | 530.83 | 625.00 | 365.52 | 630.00 |
| Women >55 years | 9 (18%) | 11 (16.2%) | 513.64 | 400.00 | 287.31 | 475.00 |

| 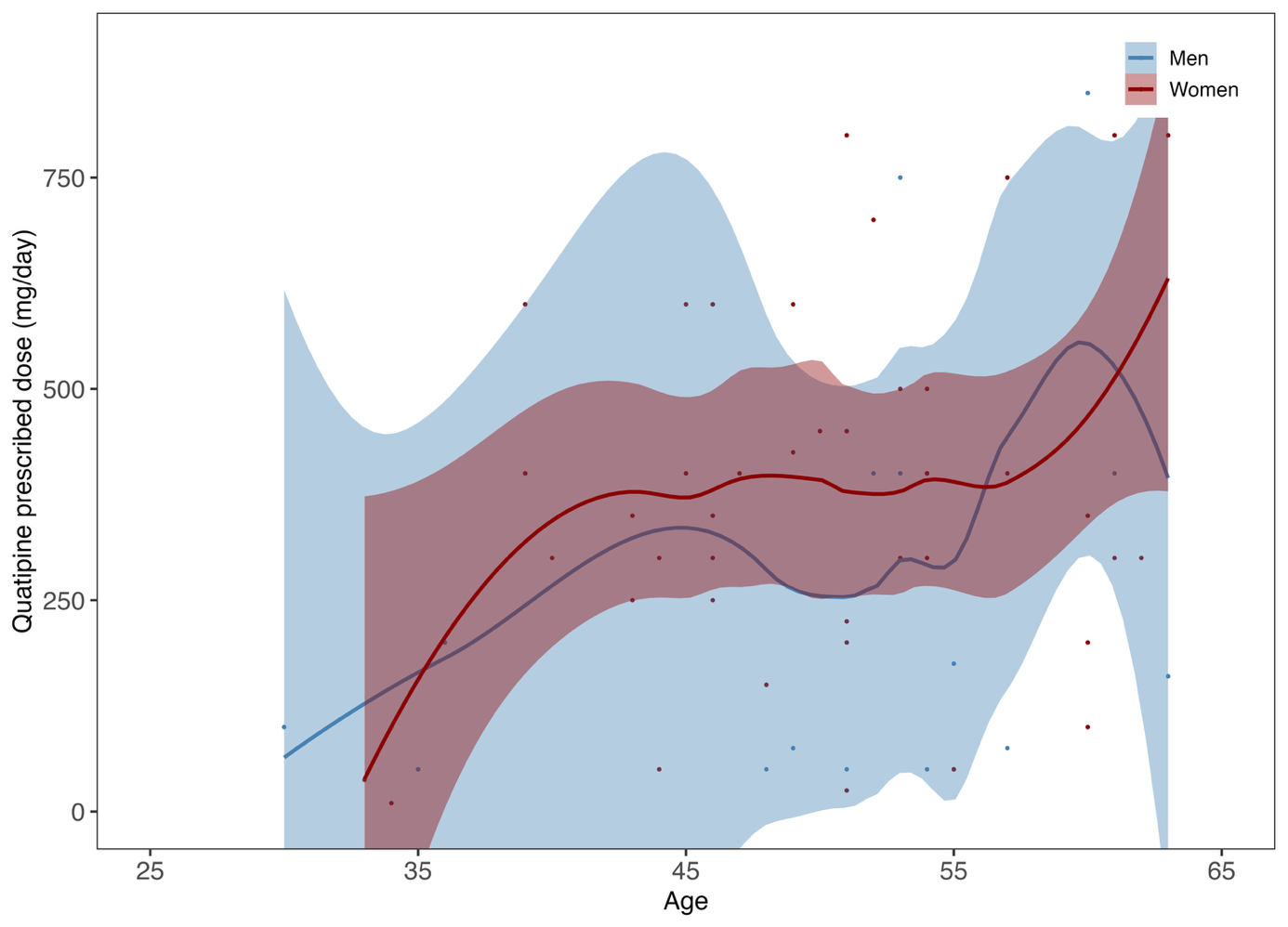 |
| --- |
| **Figure A8.** Quetiapine prescribed dose (mg/day) for men and women across ages |
